# Blood feeding by the Rocky Mountain spotted fever vector, *Dermacentor andersoni*, induces interleukin-4 expression by cognate antigen responding CD4^+ ^T cells

**DOI:** 10.1186/1756-3305-2-47

**Published:** 2009-10-08

**Authors:** Venkata D Boppana, Saravanan Thangamani, Francisco J Alarcon-Chaidez, Adam J Adler, Stephen K Wikel

**Affiliations:** 1 Department of Pathology, Center for Biodefense and Emerging Infectious Diseases and Center for Tropical Diseases, School of Medicine, University of Texas Medical Branch, Galveston, Texas, 77555, USA; 2 Department of Immunology, Center for Immunotherapy of Cancer and Infectious Diseases, School of Medicine, University of Connecticut Health Center, Farmington, Connecticut, 06030, USA

## Abstract

**Background:**

Tick modulation of host defenses facilitates both blood feeding and pathogen transmission. Several tick species deviate host T cell responses toward a Th2 cytokine profile. The majority of studies of modulation of T cell cytokine expression by ticks were performed with lymphocytes from infested mice stimulated in vitro with polyclonal T cell activators. Those reports did not examine tick modulation of antigen specific responses. We report use of a transgenic T cell receptor (TCR) adoptive transfer model reactive with influenza hemagglutinin peptide (110-120) to examine CD4+ T cell intracellular cytokine responses during infestation with the metastriate tick, *Dermacentor andersoni*, or exposure to salivary gland extracts.

**Results:**

Infestation with pathogen-free *D. andersoni *nymphs or administration of an intradermal injection of female or male tick salivary gland extract induced significant increases of IL-4 transcripts in skin and draining lymph nodes of BALB/c mice as measured by quantitative real-time RT-PCR. Furthermore, IL-10 transcripts were significantly increased in skin while IL-2 and IFN-γ transcripts were not significantly changed by tick feeding or intradermal injection of salivary gland proteins, suggesting a superimposed Th2 response. Infestation induced TCR transgenic CD4+ T cells to divide more frequently as measured by CFSE dilution, but more notably these CD4+ T cells also gained the capacity to express IL-4. Intracellular levels of IL-4 were significantly increased. A second infestation administered 14 days after a primary exposure to ticks resulted in partially reduced CFSE dilution with no change in IL-4 expression when compared to one exposure to ticks. Intradermal inoculation of salivary gland extracts from both male and female ticks also induced IL-4 expression.

**Conclusion:**

This is the first report of the influence of a metastriate tick on the cytokine profile of antigen specific CD4+ T cells. Blood feeding by *D. andersoni *pathogen-free nymphs or intradermal injection of salivary gland extracts programs influenza hemagglutinin influenza peptide specific TCR transgenic CD4+ T cells to express IL-4.

## Background

Global medical and veterinary public health importance of ticks is due to their ability to transmit a great variety of infectious agents [1-3]. Biological differences among Ixodidae (hard ticks) result in distinct clades of pathogens transmitted by different tick species [3]. Characterizing the complex interactions occurring at the tick-host interface increases understanding of pathogen transmission and establishment as well as facilitates efforts to develop novel disease prevention strategies [4].

The family Ixodidae consists of two major phyletic lines the Prostriata and the Metastriata [5]. Members of these two phyletic lines differ in reproductive biology and ecology, but both successfully adapted to obligate blood feeding life styles. *Ixodes scapularis*, a prostriate, and *Dermacentor andersoni*, *a *metastriate, are members of the two most divergent tribes of the Ixodidae [5]. Comparison of salivary gland transcriptomes of *I. scapularis *[6] and *D. andersoni *[7] revealed that they express similar broad categories of gene families whose members bear little resemblance to each other. This finding suggests that different molecules, and perhaps distinct biological strategies, are used by these evolutionarily divergent tick species to converge on the same objective of successful blood feeding and pathogen transmission. Blood feeding ticks modulate host hemostasis, wound healing, extracellular matrix, and innate and adaptive immune responses [6-8].

*Dermacentor andersoni*, Rocky Mountain wood tick, is a competent vector in nature for *Rickettsia rickettsii*, *Coxiella burnetii*, *Francisella tularensis*, Colorado tick fever virus, Powassan virus, *Anaplasma marginale*, and it is capable of inducing tick paralysis [1,3,9,10]. Host defenses are counteracted by tick saliva which contains pharmacologically active molecules essential for successful blood feeding and pathogen transmission [4,11]. *Dermacentor andersoni *modulates host immune responses and creates a potentially immunologically privileged environment for pathogen transmission and establishment by suppressing T cell proliferation; T cell production of IL-2 and interferon (IFN)-γ; reducing macrophage elaboration of tumor necrosis factor (TNF)-α and IL-1β; and, down-regulation of endothelial cell expression of ICAM-1 [12-14].

Amongst tick immunomodulatory strategies, several prostriate and metastriate species have been shown to induce Th2 cytokine polarization [4,15-17]. Many of these studies were performed with lymphocytes obtained from infested mice prior to stimulation in vitro with mitogens that are polyclonal T cell stimulators, however, rather than examining the ability of ticks to modulate antigen specific responses in vivo [4]. Studying the influence of ticks on an antigen specific T-cell response in vivo is challenging due to the low number of T cells reactive with a specific antigen [18]. That problem can be overcome using a transgenic T cell receptor (TCR) adoptive transfer model to study early antigen specific T-cell responses in vivo [19]. We recently adapted such a TCR transgenic adoptive transfer model [20,21] to study the influence of infestation with pathogen-free *I. scapularis *nymphs and salivary gland extract on CD4+ T cell cytokine production [22]. Both blood feeding by nymphs of *I. scapularis*, a prostriate tick, or intradermal injection of salivary gland extract induced significant expression of the signature Th2 cytokine IL-4 [22]. In the current study, we found that the metastriate tick *D. andersoni *can polarize antigen-specific CD4+ T cell response to a Th2 profile in a manner similar to *I. scapularis*. Given the dissimilarity in the transcriptomes between these two divergent tick species [6,7], they likely express distinct saliva molecules that induce Th2 responsiveness.

## Methods

### Mice, adoptive transfers and flow cytometry

Adoptively transferred CD4+ T cells were derived from 6.5 TCR transgenic mice expressing a transgenic TCR specific for an I-E^d ^restricted influenza virus PR8 hemagglutinin (HA) epitope (^110^SFERFEIFPKE^120^) [23] maintained on a BALB/c Thy1.1 background. Adoptive transfer recipients purchased from Jackson Laboratory were 5-6 week-old female BALB/c Thy1.2 mice. All experimental procedures involving vertebrate animals were approved by the Institutional Animal Care and Use Committee of the University of Texas Medical Branch.

Spleen and lymph node cells from 6.5 TCR transgenic mice were depleted of CD8^+ ^cells using magnetic beads (Dynabeads, Invitrogen, Carlsbad, CA), and the remaining naïve Thy1.1^+ ^6.5 TCR transgenic CD4 T cells were labeled with CFSE immediately prior to adoptive transfer into recipients that had been exposed to tick feeding or salivary gland extract (SGE), and 200 μg intradermal injection of soluble HA peptide. Subsequent functional analyses were performed as previously described [20-22,24,25]. Briefly, TCR transgenic CD4 T cells harvested from draining brachial and axillary lymph nodes (identified as CD4^+^Thy1.1^+^) on day 4 post-adoptive transfer were analyzed directly ex vivo for proliferation via dilution of CFSE.

Further, intracellular cytokine expression in divided TCR transgenic CD4 T cells was analyzed following a 5 hour re-stimulation with 100 μg/ml HA peptide plus 5 μg/ml Brefeldin A (Sigma-Aldrich, St Louis, MO). Note that the HA peptide was presented to the TCR transgenic CD4 T cells by MHC class II-expressing antigen presenting cells (dendritic cells, macrophages and B cells) that were already present in the draining lymph nodes when the single cell suspensions were prepared. Intracellular cytokine expression is determined on gated CD4^+^Thy1.1^+^CFSE^diluted ^cells. All quantitative data are expressed as the mean + SEM, and differences in cytokine expression levels between experimental groups were analyzed using ANOVA Turkey's multiple comparison test.

### Ticks and tick infestation

Pathogen-free *D. andersoni *nymphs were obtained from a colony maintained at the University of Texas Medical Branch according to previously described methods [26]. Ticks are kept at 22°C, under a 14 hour-light/10 hour-dark photoperiod in 16 ml glass vials (Wheaton Glass, Millville, NJ) with a mesh top over a super-saturated solution of potassium nitrate to provide desired relative humidity. For colony maintenance, larvae and nymphs obtained blood meals from mice and adults were fed on rabbits.

Mice receiving a single exposure to ticks were infested with five pathogen-free *D. andersoni *nymphs on days -4 and -1 followed by adoptive transfer of TCR transgenic CD4+ T cells and intradermal injection of 200 μg HA peptide (110-120) at the tick feeding site on day 0. Draining -lymph node cells were collected for analysis on day 4 after adoptive transfer. Since nymphs typically require five days to fully engorge, infestations on days -4 and -1 continuously exposed mice to tick saliva proteins prior to and during adoptive transfer and peptide administration. Effects of repeated exposure to tick feeding were assessed by a primary infestation with two applications separated by three days of five pathogen-free nymphs. Upon completion of tick feeding during the primary exposure, mice were maintained tick-free for two weeks. A second infestation consisted of five pathogen-free nymphs applied on days -4 and -1 followed by adoptive transfer of TCR transgenic CD4+ T cells and intradermal injection of HA peptide at the tick feeding site on day 0. Lymph node cells were collected for analysis on day 4 of the second infestation.

Ticks were confined within a capsule consisting of the top half of a 1.5 ml microcentrifuge tube (Fisher Scientific, Pittsburgh, PA) secured to clipped fur on the shoulder region with a four to one mixture (w/w) of calophonium (rosin, Sigma-Aldrich, St. Louis, MO) and beeswax. Engorged nymphs were removed from the capsule as they detached from the mouse. Capsules were also applied to control mice in a manner identical to experimental animals; however, these mice were not exposed to tick feeding.

### Salivary glands extract (SGE) preparation and administration

Adult male and female *D. andersoni *were fed for four days on white New Zealand rabbits [26], at which time they were removed for collection of salivary glands. Prior to dissection, ticks were washed with sterile distilled water and then surface sterilized with 70% ethanol. Salivary glands were removed and placed together into a minimal volume of 0.15 M Dulbecco's phosphate buffered saline, pH 7.2 (Gibco) held on ice. Salivary glands from males and females were pooled separately and sonicated at 55 kHz for 1 minute while held in an ice water bath followed by centrifugation at 14,000 × g for 20 minutes at 4°C. Supernatant was collected as salivary gland extract (SGE) and protein concentration determined by bicinchoninic acid assay [27]. The SGE was divided into 50 μl aliquots and stored at -30°C. All samples were frozen and thawed once, and 10 μg of SGE diluted in 50 μl sterile Hanks' buffer was administered to mice by intradermal injection.

### Real-time RT-PCR to measure cytokine gene expression

Skin punch biopsies (4 mm) were obtained from sites of tick attachment or SGE injection on day 4. Control biopsies were also obtained from a site with attached capsule without ticks and received a buffer injection. Draining lymph nodes were collected from experimental and control animals at the same time. All tissue samples were stored in RNALater (Ambion, Austin, TX) until further processing [25]. Total RNA was extracted using RiboPure™ kit (Ambion) and genomic DNA contamination was eliminated by DNAse treatment. Quality of extracted total RNA was analyzed by denaturing formaldehyde gel electrophoresis, and quantified using a NanoDrop 1000 (ThermoScientific, Pittsburgh, PA). First strand cDNA was synthesized from 1 μg total RNA using a Retroscript 1^st ^Strand cDNA synthesis kit (Ambion) and subsequently used as template for real-time RT-PCR analysis. Oligonucleotide primer sequences were previously described [25]. Real-time RT-PCR amplification was performed using RT^2^Real-Time™ SYBR Green/Fluorescein PCR master mix (BioRad) in an iCycler (BioRad, Hercules, CA). Typically, PCR was performed by heating to 95°C for 10 min to heat-activate the HotStart Taq DNA Polymerase followed by 40 cycles of 15 sec at 94°C then 60 sec at 60°C. All reactions were performed in triplicate. The *Gapdh *gene was used as a normalizing standard, and mRNA extracted from biopsies and lymph nodes of mice having capsules without tick infestation or injected with buffer (rather than SGE) were considered naïve and assigned an arbitrary value of 1.0. Changes in tick infested or SGE-induced cytokine gene expression were calculated as the ratio between tick infested or SGE and naïve samples.

## Results

### Cytokine responses induced by *D. andersoni* nymphs or SGE

**Figure 1:**
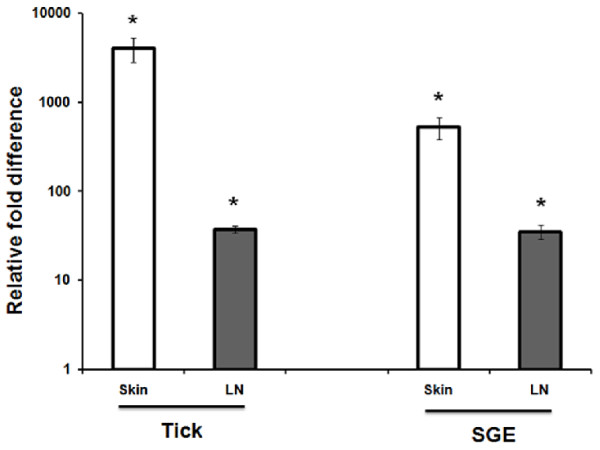
**Quantitative real-time RT-PCR measurement of tick infestation and salivary gland extract (SGE)-induced IL-4 expression in skin and draining lymph nodes**. IL-4 mRNA measured in samples isolated from skin and draining lymph nodes of uninfested (naive) mice were assigned an arbitrary value of 1.0, and IL-4 mRNA measured in experimental samples were calculated as a ratio (fold-difference) relative to naive. Asterisks indicate P < 0.001 compared to naive, and n = 3 mice per group.

To begin to characterize how the metastriate tick *D. andersoni *modulates host immune responses, we used quantitative real-time RT-PCR to measure cytokine expression at the cutaneous sites of tick feeding or salivary gland extract (SGE) injection as well as in the draining lymph nodes. Notably, expression of mRNA encoding the Th2 signature cytokine IL-4 was up-regulated 2791-fold at tick bite sites (P < 0.001) and 527-fold at intradermal injection sites (P < 0.001) of 10 μg SGE (Figure 1 and Table 1). Transcript levels of IL-4 were significantly up-regulated 37-fold (P < 0.001) in draining lymph nodes as a result of tick infestation and 36-fold following SGE injection (P < 0.001). Although less dramatic than IL-4 changes, skin levels of IL-10 mRNA were significantly up-regulated 4-fold by tick bite (P < 0.05) and 10-fold by SGE (P < 0.05) (Table 1), while neither IL-2 nor IFN-γ mRNAs were changed in response to either *D. andersoni *feeding or salivary gland proteins. Levels of IL-10 transcripts were not significantly changed in draining lymph nodes by either treatment.

**Table 1:** Relative fold-differences of Th1/Th2 cytokines in skin and lymph nodes following intradermal inoculation of Dermacentor andersoni salivary gland extract (SGE) or infestation with pathogen-free nymphs.

	**Relative fold-difference** **in skin**		**Relative fold-difference in lymph node**	
	**SGE**	**Tick**	**SGE**	**Tick**

**IL-2**	0.25 ± 0.09	0.48 ± 0.01	1.4 ± 0.1	2 ± 0.4

**IL-4**	527 ± 147*	2791 ± 504*	35 ± 6.3*	37 ± 3.4*

**IL-10**	10 ± 1.3**	4 ± 0.7**	0.7 ± 0.1	0.4 ± 0.07

**IFN-γ**	3.4 ± 0.4	0.8 ± 0.2	0.4 ± 0.09	1.2 ± 0.19

* Significant at P < 0.001

** Significant at P < 0.05

### *Dermacentor andersoni* infestation programs CD4+ T cells to express IL-4

**Figure 2:**
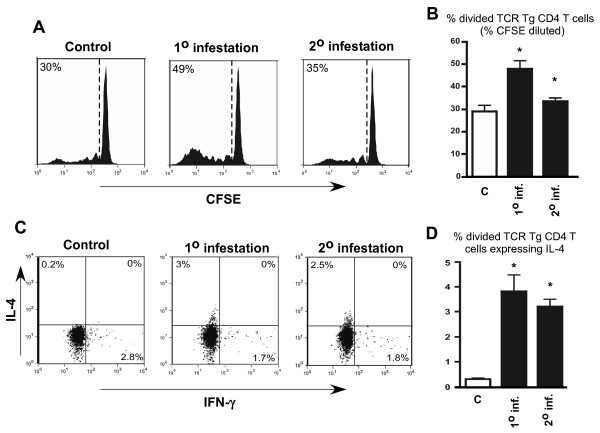
**Infestation with pathogen-free *Dermacentor andersoni *nymphs programs adoptively transferred HA-specific TCR transgenic CD4+ T cells to express IL-4**. Panel A shows the percentage of divided cells in representative CFSE-dilution histograms for uninfested controls, mice receiving a primary (1°) infestation or a repeated infestation (2°) four weeks after an initial exposure to tick feeding. Panel B represents the mean percent ± standard error of CFSE labeled TCR transgenic CD4 T cells. Panel C depicts representative intracellular staining for IFN-γ vs IL-4 expression. Panel D presents quantitative analysis of cytokine expression. For panels B and D, asterisks indicate P < 0.05 compared to uninfested controls. N = 4 mice per group.

Since the above experiment measured cytokine mRNAs in whole skin biopsies and un-fractionated lymph nodes samples, the measured *D. andersoni *tick-induced alterations in cytokine expression patterns where likely a composite of changes induced in both T cells and innate immune cell types. To examine changes in cytokine expression in CD4 T cells specific for antigens introduced at the *D. andersoni *tick-feeding site, we utilized a TCR transgenic adoptive transfer system that we previously developed to examine the response to *I. scapularis *[22]. TCR Tg CD4 T cells that recognize a class II-restricted epitope deriving from influenza hemagglutinin are adoptively transferred into recipient mice that have been infested with tick nymphs, and a bolus of soluble peptide corresponding to the cognate HA epitope is injected intradermally at the site of tick attachment. Because the HA peptide does not possess intrinsic adjuvant activity, when introduced into control non-infested mice it induces the cognate CD4 T cells to proliferate without differentiating into discrete Th phenotypes (i.e., Th1 or Th2), thus allowing the influence of tick infestation to be readily detected. Consistent with our previous studies [21,22] ~30% of the adoptively transferred TCR CD4+ T cells recovered from the lymph nodes draining the peptide injection site underwent proliferation indicated by dilution of the fluorescent tracking dye CFSE (Figure 2A and 2B) in the absence of tick infestation. Infestation with pathogen-free *D. andersoni *nymphs on days -4 and -1 prior to adoptive transfer and intradermal administration of HA peptide resulted in a significant increase in CD4+ T cell proliferation to ~50% (P < 0.001) (Figure 2A and 2B). When a second infestation occurred 14 days after primary exposure to ticks, CFSE dilution was reduced slightly compared to the single infestation (P < 0.01) yet remained greater than non-infested controls (P < 0.05).

Tick infestation also programmed the TCR transgenic CD4 T cells to express IL-4 as measured by intracellular flow cytometry staining following in vitro re-stimulation with cognate HA peptide. As expected, TCR transgenic CD4+ T cells recovered from draining lymph nodes of control non-infested mice expressed background levels of IL-4 (0.2% of total divided cells) (Figure 2C and 2D). Primary tick infestation induced approximately 3% of divided cells to express IL-4 cytokine. Although IL-4 expression was modest, it was highly reproducible and significant (P < 0.001). Additionally, the percentage of CD4+ T cells expressing IL-4 expression is similar to what we previously observed during infestation with the prostriate tick *I. scapularis *[22]. A second infestation separated from the first exposure to ticks by 14 days, resulted in approximately 2.5% of CD4 T cells expressing IL-4. Similar to primary infestation, IL-4 expression programmed by secondary infestation was also significantly increased over un-infested controls (P < 0.05). IFN-γ expression in TCR transgenic CD4+ T cells recovered from control uninfested recipients was minimal, consistent with the lack of Th1 adjuvant activity in the soluble HA peptide preparation [21,22], and neither primary nor secondary tick infestation impacted expression of this Th1 cytokine (Figure 2C).

### Salivary gland extract contains IL-4 programming activity

**Figure 3:**
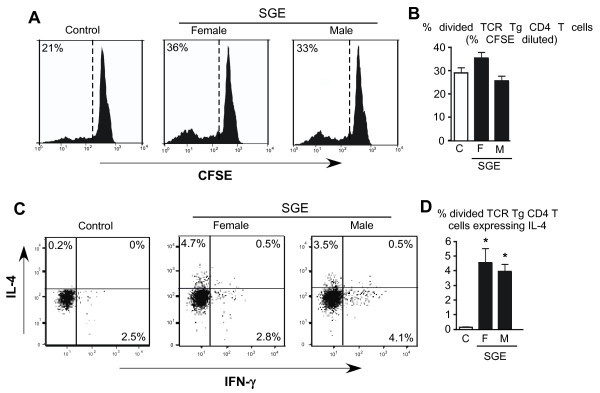
**Intradermal injection of female or male *Dermacentor andersoni *derived salivary gland extract (SGE) programs TCR Tg CD4+ T cells to express IL-4**. Panel A, representative histograms of CFSE dilution. Panel B, quantitation of CFSE dilution. Panel C depicts representative intracellular staining for IFN-γ vs IL-4 expression. Panel D, quantitation of cytokine expression. For panels B and D, asterisks indicate P < 0.05 compared to uninfested controls. N = 4 mice per group.

To assess whether the *D. andersoni *IL-4 programming activity is mediated via a soluble salivary factors, salivary gland extract (SGE) was administered by intradermal injection. Similar to tick feeding (Figure 2), male and female SGE programmed adoptively transferred TCR transgenic CD4+ T cells to express IL-4 (P < 0.05) but not IFN-γ (Figure 3C and 3D). Dilution of CFSE was not impacted (Figure 3A and 3B). Real-time RT-PCR analysis indicated that similar to tick feeding, intradermal injection of SGE induced expression of IL-4 mRNA in the skin (P < 0.001) and draining lymph nodes (P < 0.001) as well as increased IL-10 mRNA in the skin (P < 0.05) (Figure 1 and Table 1).

## Discussion

Ticks and infectious microbes both modulate host immune defenses to enhance their likelihood of survival [4,28-30], and tick-borne pathogens exploit the modified host environment to facilitate transmission and establishment within the host [4,11,31]. Depending upon life cycle stage, a tick can remain attached and consume blood from a host for approximately four days to over two weeks [32]. In order to facilitate this prolonged, continuous and intimate contact with the host, tick salivary glands produce a complex array of biologically active molecules that modulate host hemostasis, pain/itch responses, wound healing, tissue remodeling and immunity [6,7]. The highly divergent salivary gland transcriptomes of a prostriate, *I. scapularis*, and a metastriate, *D. andersoni*, suggests that these two species utilize different repertoires of biologically active molecules to achieve the same ends of successful blood feeding and pathogen transmission [6,7,31].

Acquired host resistance to *D. andersoni *is mediated in part by antibodies, complement, and cell mediated immunity [33-35]. Not unexpectedly, *D. andersoni *modulates host responses that have the potential to reduce successful parasitism. In vitro proliferative responses of T cells were reduced in response to infestation with *D. andersoni *[12], and a salivary gland protein, p36, that inhibited mitogen driven T cell proliferation was subsequently cloned, expressed and characterized [36]. Salivary gland extracts prepared during the course of engorgement of *D. andersoni *females suppressed T-cell production of interleukin (IL)-2, interferon (IFN)-γ, macrophage elaboration of tumor necrosis factor (TNF)-α, and IL-1β [13]. Host epidermal Langerhans cells trap *D. andersoni *salivary gland antigens [37] and these dendritic cells are essential for development of host immune responses to tick bite [38]. Expression of the endothelial cell adhesion molecule ICAM-1 is significantly down-regulated by *D. andersoni *salivary gland extract, likely reducing the inflammatory response to tick bite [14].

Amongst host immune responses modulated by ixodid ticks, T cell cytokine polarization to an up-regulated Th2 profile appears to be a common strategy often accompanied by a decrease in Th1 cytokine expression [4]. Most studies used tick salivary gland extracts, tick saliva or mitogens to induce in vitro cytokine production by splenocytes or lymph node cells from tick infested animals or similar cell populations from animals never exposed to ticks [4,15,16,39,40]. These studies provided a global evaluation of T cell responses, but they did not allow for assessment of how ticks influence antigen-specific T cell responsiveness. We recently demonstrated, using the TCR transgenic adoptive transfer model used in the current study, that blood feeding by the prostriate tick, *I. scapularis*, significantly increases the number of CD4 T cells responding to bite site-associated antigen that can express IL-4 [22]. In contrast to most studies examining polyclonal T cell responses to ticks or tick derived molecules [4], the increase in IL-4 expression was not accompanied by reduced expression of the Th1 cytokine IFN-γ [22]. A Th2 response was superimposed on the background Th1 response.

In the present study, infestation with the metastriate tick, *D. andersoni*, induced CD4+ T cell expression of IL-4 at levels comparable to that stimulated by feeding of *I. scapularis *nymphs [22]. This observation supports the idea that Th2 polarization occurs widely amongst ticks, since *D. andersoni *and *I. scapularis *represent the two most divergent tribes of the family Ixodidae [5,41]. CD4+ T cells begin to express IL-4 transcripts within 30 minutes of stimulation under Th2 polarizing conditions and to express IL-4 by 48 hours with as many as five to six cell divisions by the fourth to fifth days post-stimulation [42].

*Dermacentor andersoni *also induced expression of IL-4 in skin. The cellular source of IL-4 may be mast cells or cutaneous basophils rather than CD4+ T cells, although IL-4 produced by these innate immune cell types could potentially program the CD4+ T cells in draining lymph nodes to express IL-4 [43-47]. IL-10 was also induced in skin, but not in draining lymph nodes, although the significance of this expression pattern is not presently clear. Nevertheless, Th2 programming by these two tick species is likely mediated via distinct factors given the dissimilarities between their respective transcriptomes [6,7]. Molecule(s) have not been identified that are responsible for Th2 polarizing activity in *D. andersoni *saliva. Recently, we identified a novel sphingomyelinase-like activity in the saliva of *I. scapularis *that is capable of inducing IL-4 expression in the adoptive transfer model system used in this study. In silico comparisons of the salivary gland transcriptomes for *I. scapularis *[6] and *D. andersoni *[7] did not reveal the presence of a sphingomyelinase-like homologue in *D. andersoni*. Thus, *D. andersoni *likely expresses unique Th2 polarizing factors.

In the current study, multiple infestations with *D. andersoni *nymphs reduced the proliferative response of specific CD4 T cells, consistent with previous studies examining polyclonal T cell responses during in vitro recall assays [11,48]. This observation could be suggestive of the ability of the host to develop a mechanism in response to infestation that blunts the activity of the tick salivary factors that modulate T cell proliferation. In addition, the ability of *D. andersoni *infestation to program specific CD4 T cell expression of IL-4 was significantly reduced during the second infestation. This finding is consistent with that observed in response to infestation with *I. scapularis *nymphs where IL-4 polarizing potential was partially reduced during secondary infestation [22].

Both male and female salivary gland extracts of partially fed *D. andersoni *contained the ability to program IL-4 expression in specific CD4+ T cells. Similarly, both male and female *D. andersoni *salivary glands produce p36, a protein inhibitor of T cell proliferation [49]. In addition to the need to blood feed, the reproductive biology of metastriate ticks provides a logical basis for salivary glands of both sexes to contain a repertoire of biologically active molecules to modify host defenses. Males attach to the host first and emit pheromones that attract the female for mating at the feeding site [5]. Essentially, the male prepares a "privileged" site for the female to attach and blood feed. Her feeding then further contributes to modulation of host defenses.

## Competing interests

The authors declare that they have no competing interests.

## Authors' contributions

VDB, ST and SKW conceived the experiments. VDB and ST performed experiments. VDB, ST, FAC, AJA and SKW analyzed data and prepared the manuscript.
